# Prevention and health promotion regarding sexually transmitted infections (STI) among university students in Germany

**DOI:** 10.1007/s10389-023-01876-7

**Published:** 2023-04-25

**Authors:** Pascal Voegele, Wolf Polenz

**Affiliations:** Department of Health Sciences, Faculty Life Sciences, University of Applied Sciences Hamburg, Ulmenliet 20, 21033 Hamburg, Germany

**Keywords:** Sexually transmitted infections, STI prevention, Students, University, Pre-exposure prophylaxis, Antiretroviral therapy, Self-efficacy

## Abstract

**Aim:**

University students are sexually active, and the sexual risk behavior of this group is higher than that of the general population. The prevention of sexually transmitted infections (STIs) emphasizes the need for comprehensive knowledge about behaviors for STI protection and the actual realization of these behaviors.

**Subject and methods:**

First, an online questionnaire was developed to record the knowledge and realization of STI-protective behaviors among students at Hamburg University of Applied Sciences (HUAS) to conduct quantitative cross-sectional interviews. The sample included 1532 students. Specific aspects of the interview are based on lower response rates. The correlations were then tested by Spearman’s rank correlation coefficient and Pearson’s chi-squared test.

**Results:**

Significant positive correlations were identified between the self-efficacy (SE) and the use of condoms, STI vaccinations, STI tests, and HIV pre-exposure prophylaxis (PrEP). Significant negative correlations were suggested between substance use and the use of condoms and the use of PrEP and the intake of antiretroviral therapy (ART). Significant positive correlations were identified between the knowledge about STI-protective behaviors and the usage of STI-protective vaccinations, STI tests, and ART. Significant positive correlations were identified between the experiences in terms of STIs and the knowledge about STI-protective vaccinations, use of PrEP, and use of ART.

**Conclusion:**

Moreover, the results indicate that students with a divergent sexual identity have a higher level of knowledge about STI-protective behaviors. The sexual health of university students should be improved by preventive measures to improve the sexual health of individual students and their social environments.

**Supplementary Information:**

The online version contains supplementary material available at 10.1007/s10389-023-01876-7.

## Introduction

The number of university students changing sexual partners is higher than that of the general population. University students are also in a specific life stage between their youth and adult life. This life stage is characterized by trying new roles, including in terms of their own sexuality (Matthiesen et al. 2017). This is associated with specific sexual risk behaviors because of the accompanying sexual practices. Combined with the higher number of sexual partners, university students are more likely to contract sexually transmitted infections (STIs), which also implies a risk for their sexual partners (Riemenschneider et al. 2017). The age group of university students is also often affected by STIs which are asymptomatic, additionally increasing the risk of transmission (Weissenbacher and Weissenrieder 2006). Against this background, STI-protective behaviors seem to be necessary. In the context of this article, in addition to condoms, STI-protective behaviors include HIV pre-exposure prophylaxis (PrEP), vaccinations against STI, and the use of STI tests. Because of the secondary protection from HIV for sexual partners, effective HIV therapy is also an STI-protective behavior [17]. This bandwidth of STI-protective behaviors is rarely considered in German sexual health studies. The knowledge of behaviors which protect from STIs is also largely not illuminated in these studies (Haversath et al. 2017; Hinzpeter et al. 2022; Riemenschneider et al. 2017). The aim of this study was to increase the knowledge and realization of STI-protective behaviors among students at the NN University of Applied Sciences (NNUAS), which is necessary in order to derive recommendations for actions that are suitable for the target group.

## Method

### Sampling

The online questionnaire was sent to all students of NNUAS in German. Students from all faculties (Life Sciences, Economics & Society, Technology & Informatics and Design, Media & Information) were addressed. The participants had no time limit and were not rewarded. The participation was voluntary and under conditions of anonymity.

### Questionnaire

Based on several established questionnaires, a new questionnaire was developed specifically for this study, which contains predominantly closed items and measures different constructs. A five-point Likert scale was formulated for measuring the self-efficacy (SE). The Condom Use Self-Efficacy Scale (Brafford and Beck 1991), European MSM Internet Survey (ESTICOM 2017), HIV Testing Self-Efficacy Scale (Zhao et al. 2018), and the HIV Adherence Self-Efficacy Scale (Johnson et al. 2007) were used for measuring SE and realization in terms of single STI-protective behaviors. Also, sociodemographic variables, especially sexual identity because of the accompanying sexual practices and risk factors (Riemenschneider et al. 2017), and relationship status were measured (Schutte 2017). The use of alcohol and other illegal substances was captured by the Youth Risk Behavior Survey (CDC 2021). The questionnaire was pretested in three steps. The first step was a qualitative pretest with a sample of seven students. The questionnaire was adjusted after this step. Test statistical analyses were performed in the third step of the pretest.

### Statistical analysis

The data were analyzed using IBM SPSS version 26.0 software. Significance was considered for *p*-values of < 0.05 and < 0.01. Scores were assigned to the items which measured the SE of single STI-protective behaviors. Scores between 1.00 (“fully agree”) and 2.00 (“agree”) were associated with high SE. Scores between 2.00 and 3.00 (“undecided”) were associated with moderate SE. Values between 3.00 and 4.00 (“disagree”) and 3.00 and 5.00 (“strongly disagree”) were associated with low SE (Brafford and Beck 1991).

Spearman’s rank correlation coefficient (ρ) was used for analyzing the correlation between the SE and the realization of STI-protective behaviors, substance use and realization, knowledge of STI-protective behaviors and their realization, and pre-experiences in terms of STIs and knowledge of STI-protective behaviors. Linear regression analyses were used for analyzing the interrelationships between SE and realization and between knowledge and realization. Pearson’s chi-squared test was used for evaluating the correlation between the sexual identity and the knowledge about STI-protective behaviors.

## Results

### Description of the sample

**Fig. 1:**
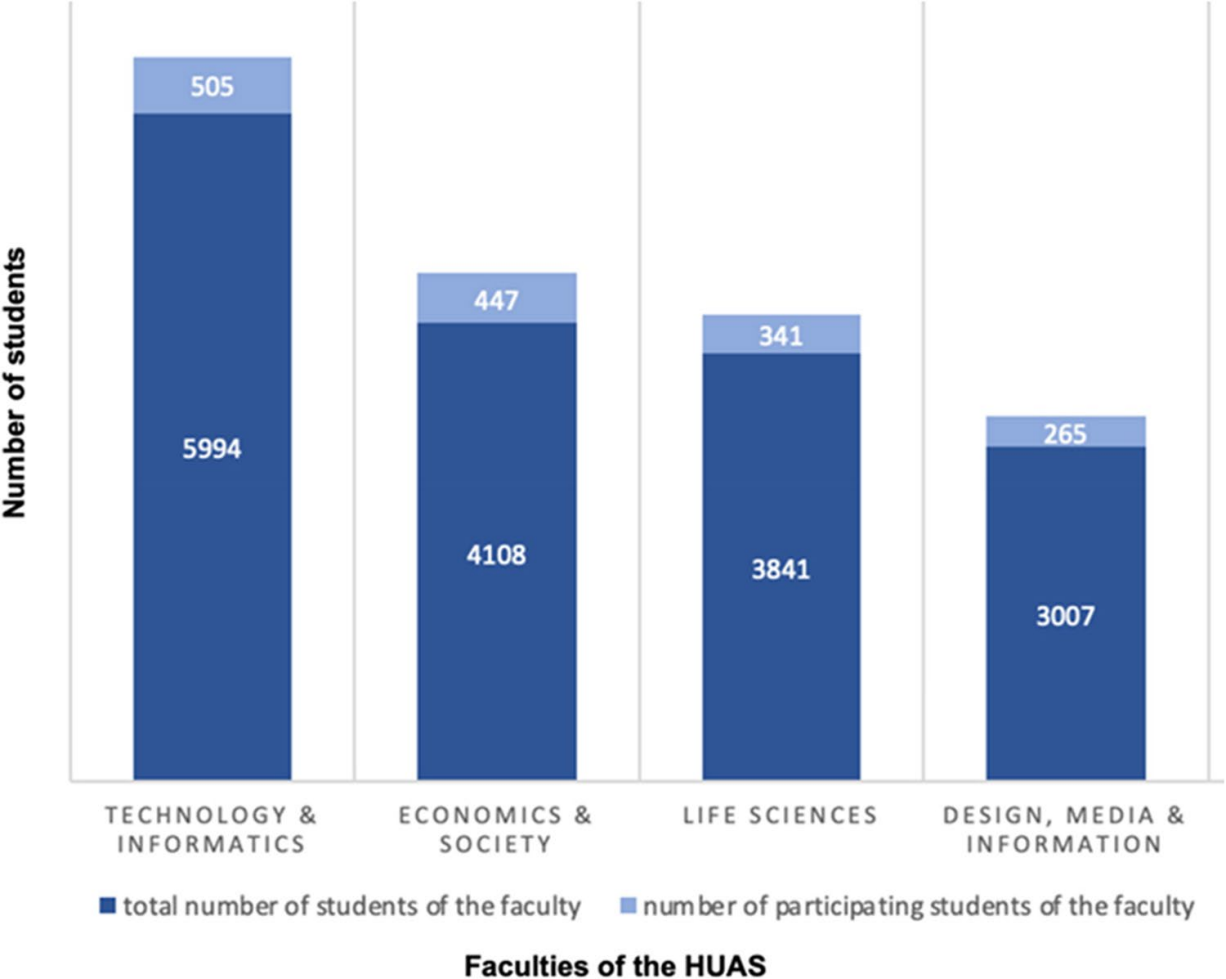
Numbers of participating students and total numbers of students of each faculty

A total of 1558 students of HUAS took part in the survey, which represents 9.3% of the population (population: 16,950). The interviews from 1532 participants could be analyzed. Among participants, 81.7% were bachelor students. Figure 1 shows the number of participating students in relation to the total number of students of each faculty.

**Fig. 2:**
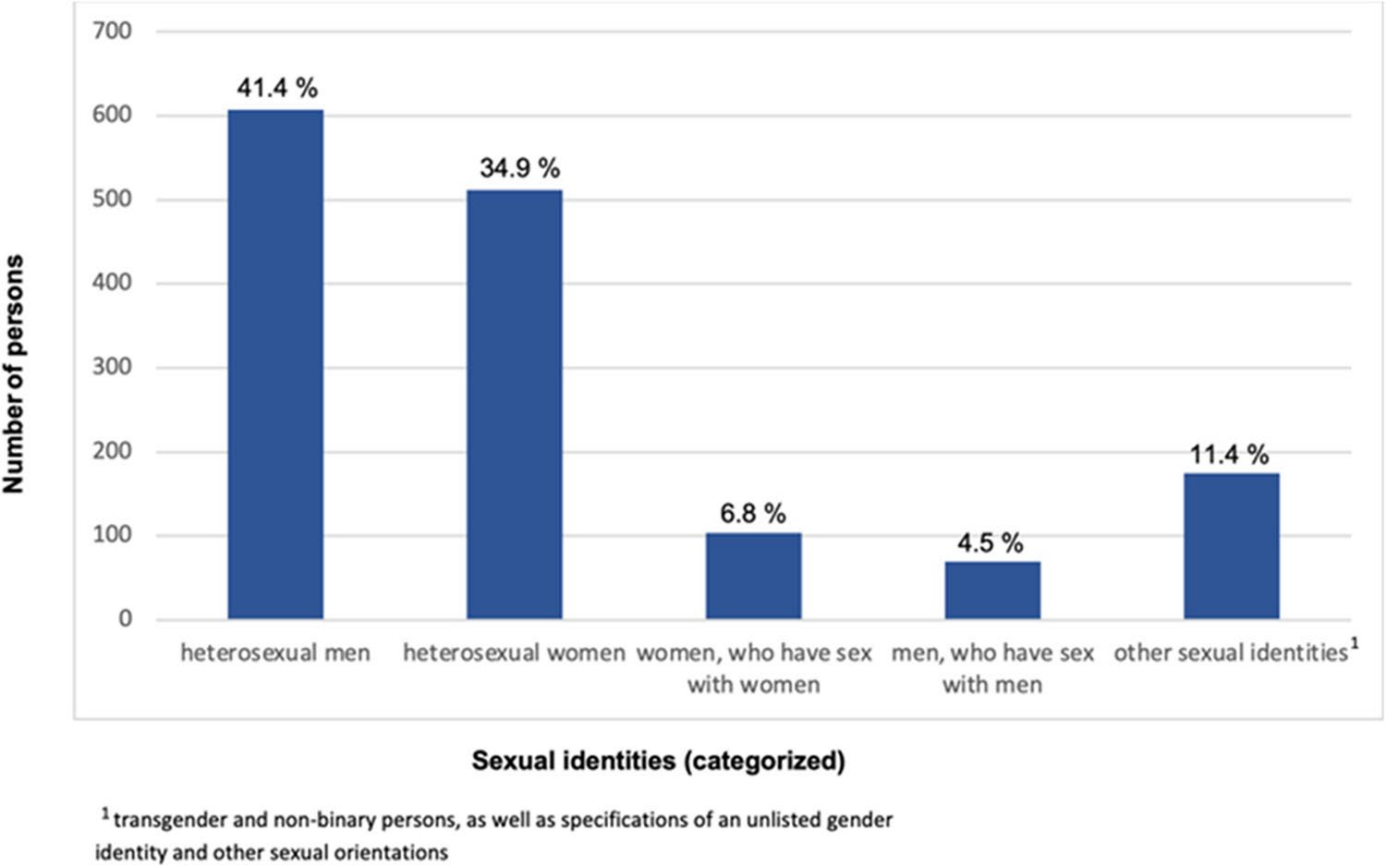
Frequencies and percentages in terms of the participants’ sexual identity

The average age was 24.8 years; 57.9% were aged 17–24 years, 28.9% were aged 25–29 years, 12.0% were aged 30–40 years, and 1.2% were older than 40. Most of the participants indicated that they were currently in a monogamous relationship (53.8%), 36.2% were single, and 5.2% reported being unsure of their relationship status. Alternative types of relationships (e.g., open partnerships) were the exception, at 4.8%. The frequencies of the participants’ sexual identity are shown in Fig. 2. The sexual identity was categorized by the reported gender identity and sexual orientation. Sexual identity was differentiated so that students with divergent gender identity or sexual orientation would feel addressed, which would increase their willingness to participate. Another reason is that sexual identity conditions sexual behavior and thus the risk for STIs because of the accompanying sexual practices (Matthiesen et al. 2017).

### Sexual risk behavior

Most of the participants reported having had one sexual partner during the past 12 months. The descriptive view of this feature suggests that differences in terms of the number of sexual partners are dependent on sexual identity. Table 1 depicts the frequencies of the number of sexual partners during the past 12 months by categories and grouped according to sexual identity. The term cis-gender describes a person who identifies with their sex assigned at birth.

**Table 1:** Frequencies and percentages of the categorized number of sexual partners during the past 12 months, grouped by sexuality

Categorized Number of sexual partners during last 12 months	Frequency	Proportion in %^1^
Heterosexual identity		
0	159	14.6
1–3	830	76.0
4-10	80	7.3
10+	23	2.1
Divergent sexual identity^2^		
0	29	8.8
1–3	234	71.1
4–10	47	14.3
10+	19	5.8
		N_complete_ =1532

^1^Proportion of the subgroup in terms of each listed sexual identity

^2^Persons who define themselves not as cis-gender and/or not as heterosexual

### Substance use

Concerning substance use, 55.1% of the participants reported having drunk alcohol before or during sexual contact within the past 6 months; 78.3% of these participants indicated this consumption as rare. The consumption of illegal substances before or during sexual contact within the past 6 months was reported by 14.9% of the participants; 9.9% of this substance use was cannabis, and 76.7% of these participants indicated rare frequency of illegal drug use during sexual contact, while 14.3% reported consumption mostly in terms of sexual contact.

The analyses of the relationship between substance use and realization of STI-protective behaviors were not significant with regard to all mentioned STI-protective behavior. Consequently, only certain behaviors could be considered. The analysis per rank correlation showed a significant (*p* < 0.05) weak negative correlation between alcohol use and the use of condoms (*ρ* = −.114) and ART (*ρ* = −.072). The calculated rank correlations of the analysis between illegal substance use and realization indicated a significant (*p* < 0.05) negative correlation between illegal substance consumption and the use of PrEP (*ρ* = −.122) and ART (*ρ* = −.155).

### STI-protective behavior

#### Realization

As a realization of STI-protective behaviors, 38.4% of the participants reported always using condoms as protection from STI, while 29.1% reported using condoms most of the time. Regarding the use of STI tests, 46.8% of the participating students indicated that they had never been tested for STIs, while 15.2% claimed to have had an STI test during the past 12 months and 22.7% more than 12 months ago. Table 2 illustrates the percentages of participants who reported being fully vaccinated and those who were unsure about their vaccination status in terms of vaccinations against hepatitis A (HAV), hepatitis B (HBV), and human papillomavirus (HPV).

**Table 2:** Percentages of participants who reported being fully vaccinated and those unsure of vaccination status in terms of vaccinations against STIs

Vaccination	Percentage of fully vaccinated participants	Percentage of participants who were not sure about their vaccination status
HBV	54.9	29.6
HAV	44.6	35.7
HPV	25.1	37.1
		N_complete_ =1558

The realization of PrEP is at 1.0%, which corresponds to 12 participants, of which six persons took PrEP daily. Of the 98.6% of the participants who reported that they did not take PrEP, 20.7% indicated that they did not do so because of no substantial HIV risk. Seventeen participants (1.4%) reported ever having used the protection of ART. Twelve of these participants (1.0%) reported ever having sex with people who take ART. Five persons (0.4%) took ART themselves.

#### Knowledge

**Fig. 3:**
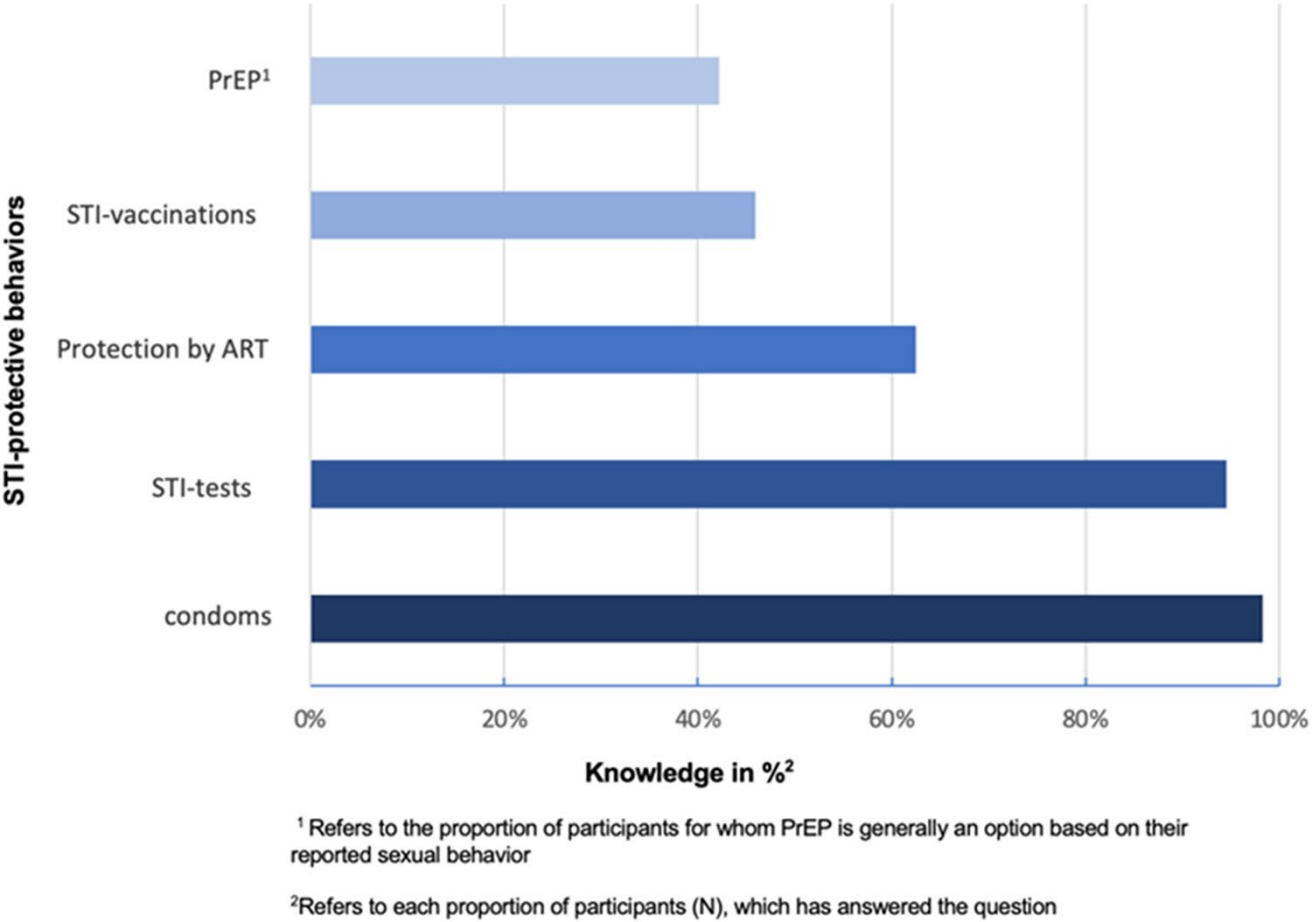
Frequencies and percentages in terms of the participants’ reported knowledge about STI-protective behaviors

The participants’ knowledge about STI-protective behaviors is shown descriptively in Fig. 3, which represents the frequencies and percentages of the samples who claimed to know each behavior.

The correlation between the knowledge about STI-protective behaviors and the realization of specific behaviors was analyzed by linear regression analysis, and Cohen’s effect sizes *f* (Cohen 1992) were calculated. A category which includes the knowledge about all listed STI-protective behaviors was created for this purpose. PrEP and condom use were not included because the regression model was not significant in general. In terms of the other STI-protective behaviors, significant correlations were found. The effect sizes of the correlations between knowledge and realization of HPV vaccination (*f* = .301) and STI tests (*f* = .250) indicate effects of medium strength. The influence of knowledge on the realization of HAV vaccination (*f* = .099), HBV vaccination (*f* = .160), and ART (*f* = .109) can be interpreted as small.

Spearman’s rank correlation coefficient was calculated in terms of the correlations between pre-experience with STIs and single STI-protective behaviors. The correlations in terms of STI tests and condom use were not significant (*p* > 0.05). The suggested correlations between pre-experiences and ART (*r* = .105), PrEP (*r* = .113), and STI vaccinations (*r* = .144) can be interpreted as weak, and also, in terms of all listed STI-protective behaviors (*r* = .130).

The correlation between heterosexual and divergent sexual identity and knowledge about STI-protective behaviors was analyzed in a first step using Pearson’s chi-squared test, from which statistical significance was determined (heterosexual identity: *p* < 0.01; divergent sexual identity: *p* < 0.05). The second step entailed the calculation of Cramer’s V and the contingency coefficient (CC) between knowledge about STI- protective behaviors and sexual identity, which is shown in Table 3.

**Table 3:** Calculated Cramer’s V and contingency coefficient values for analyzing correlations between knowledge about STI-protective behaviors and sexual identity

Sexual identity	Cramer’s V	CC	N
Heterosexual	−0.216	−0.211	1169
Divergent	−0.235	−0.499	348
N_complete:_ 1517			

The calculated values of Cramer’s V indicated very strong effects. The values of the calculated CC can be interpreted as medium-strong correlations between the knowledge about STI-protective behaviors and sexual identity.

#### Self-efficacy

The measured SE in terms of single STI-protective behaviors is shown in Table 4. Lower scores are associated with higher SE. The self-efficacy in terms of ART could not be analyzed because the sample of ART users was too small.

**Table 4:** Mean values, standard deviations, and variances of the calculated self-efficacy scores in terms of each STI-protective behavior

STI-protective behavior	Mean values of the scores	Standard deviation	Variance	*N*
Condom use	1.52	0.61	0.36	1263
STI vaccinations	1.78	0.76	0.57	1253
STI tests	1.87	0.68	0.46	1232
PrEP	2.31	0.84	0.71	956

We also analyzed the correlation between SE and the realization of single STI-protective behaviors. The correlation between SE and the use of condoms, STI vaccinations, STI tests, and PrEP was weak (ρ < .30) but significant (*p* < 0.01). Therefore, a linear regression analysis was performed with the associated effect sizes *f*. The lowest value was found in terms of PrEP (*f* = .132). The correlation between SE and the realization of STI-vaccinations was indicated as medium strong regarding HPV vaccination (*f* = .212). In terms of hepatitis A (*f* = .139) and B (*f* = .157) lower values were found. The effect sizes of the correlation between SE and using condoms (*f* = .209) and STI-tests (*f* = .239) can be interpreted as medium strong. Another regression analysis also indicated an interdependency between SE and realization of STI-protective behaviors. The effect of the realization on SE seems smaller than inverted.

## Conclusion

The most relevant risk factor of students’ sexual behavior is the number of changing sexual partners. Most participants reported 1–3 sexual partners during the past 12 months. This comparatively small number could be related to the COVID-19 pandemic. Most of the interviewed students reported having drunk alcohol at least once before or during sexual contact. This should be discussed in terms of a limited realization of STI-protective behaviors. The small number of participants who reported never having had an STI should be interpreted considering that almost half of the participants said that they had never had an STI test. Past STI could have remained undetected. Just a few participants reported using PrEP or ART for STI protection. This could be due to several factors. These STI-protective behaviors were indicated as less known by the participants. The small number of PrEP users could be due to the small number of participants who defined themselves as men who have sex with men (MSM), which represents the main target group for PrEP (RKI 2021). In terms of the secondary protection effect of ART, it could be that participants had unknowingly had sex with persons taking ART.

In general, a discrepancy becomes visible between the comprehensive knowledge and actual realization of STI-protective behaviors. This might be due to established barriers (e.g., social desirability) or substance use.

It can be assumed that SE has a significant influence on the use of considered STI-protective behaviors because of the significant correlation coefficients of each regression analysis. Previous studies support this association in terms of each listed STI-protective behavior (Baele et al. 2001; Golub et al. 2019; Ma et al. 2006; Mangi et al. 2019; Martin-Smith et al. 2018; Mateske et al. 2013; Petrovic et al. 2011). Rank correlations describe correlations between knowledge and realization of STI vaccinations, STI tests, and ART. The following regression analysis indicates that increasing knowledge results in increased realization of STI-protective behaviors. This is to be assumed especially in the case of STI vaccinations and STI tests because these methods assume explicit information. The weak correlations between STI pre-experiences and realization of STI vaccinations, PrEP, and ART should be discussed in view of the small number of participants who had received an STI diagnosis in the past. Regarding the correlations between sexual identity and knowledge about STI-protective behaviors, the consideration focuses on the differences in values determined for participants with heterosexual or divergent sexual identity. The analysis indicates that participants with divergent sexual identities have a higher level of knowledge about STI-protective behaviors. This finding is supported by published studies (Trucchi et al. 2020; Voyiatzaki et al. 2021). This could be due to the accompanying sexual practices.

It is important to consider the results of the study in the context of limitations. While a large number of heterosexual students participated, a lower response of participants with divergent sexual identities (especially MSM) was obtained. It is conceivable that participants did not participate or skipped questions because they were uncomfortable with the research topic. The capture of the SE with a Likert scale bears the risk that participants reported erroneously better values.

The study clarifies the diverse sexual risk and prevention behaviors of HUAS students. Therefore, preventive measures should contain the diversity of STI-protective behaviors and should not focus solely on the use of condoms. The broad information about STI-protective behaviors, especially STI tests, could contribute to better accessibility of the student target group.

## Supplementary information

ESM 1 (PDF 559 kb)

## Data Availability

Data available on request as SPSS data file

## Code availability (software application or custom code)

Code available on request as SPSS syntax file
