# Supplementary material for: Prevention and health promotion regarding sexually transmitted infections (STI) among university students in Germany
Source: Z Gesundh Wiss. 2023 Apr 25:1–7. Online ahead of print. doi: 10.1007/s10389-023-01876-7 (PMC10125854; doi:10.1007/s10389-023-01876-7)
Supplement: Supplementary file 1 — (PDF 559 kb) [file 10389_2023_1876_MOESM1_ESM.pdf]

Einleitung:

Anhand der vorliegenden Checkliste können Forschende eine Selbst-Evaluierung im Hinblick auf ethisch relevante Aspekte Ihres Forschungsvorhabens vornehmen. Die nachfolgenden Fragen dienen der Orientierung, ob ein Antrag auf Stellungnahme der Ethikkommission gestellt werden sollte. Falls eine oder mehrere Fragen unter den Punkten 2 bis 11 mit **JA** beantwortet werden, sollte die/der Forschende einen Antrag auf Stellungnahme an die Ethikkommission der HAW Hamburg stellen. Die Selbst-Evaluierung ersetzt kein Votum durch die Ethikkommission. Es wird empfohlen, die ausgefüllte Selbst-Evaluierung zu der Dokumentation des Forschungsvorhabens zu nehmen.

Grundsätzlich prüft die Ethikkommission nur Anträge, die VOR Beginn der Forschungsarbeiten gestellt werden. Anträge, die nachträglich eingereicht werden, können nicht berücksichtigt werden.

Falls nach dieser Selbst-Evaluierung kein vollständiger Ethikantrag erforderlich ist und es sich im Nachhinein herausstellt, dass eine Bestätigung der Ethikkommission hierüber, z.B. für eine Publikation, benötigt wird, kann die Checkliste zusammen mit einer Kurzbeschreibung des Forschungsvorhabens der Kommission vorgelegt und um Bestätigung gebeten werden.

1. Ethische Prüfung

Frage	JA	NEIN
a) Ist das Forschungsvorhaben bereits bei einer anderen Ethikkommission in Deutschland zur Begutachtung eingereicht worden?<br>⇨ Ja: **keinen** Ethikantrag stellen	☐	☒
b) Wünscht der Mittelgeber ein Ethikvotum?<br>⇨ Ja, Ethikantrag stellen	☐	☒
c) Wird für eine beabsichtige Publikation ein Ethikvotum benötigt?<br>⇨ Ja, Ethikantrag stellen	☐	☒

2. Forschung an und mit Menschen

2.1. Allgemeines

	JA	NEIN
a) Liegt **keine** freiwillige informierte Einwilligung[1] zur Studienteilnahme vor?	☐	☒
b) Behandelt das Forschungsvorhaben **keine** reine (nicht-interventionelle) Beobachtung von Menschen?	☐	☒
c) Werden in individuellen Interviews/Fragebögen oder Gruppeninterviews /-fragebögen des Forschungsvorhabens Themen angesprochen, die sensibel, peinlich oder übergriffig sind oder als stigmatisierend wahrgenommen werden können?	☒	☐
d) Können im Rahmen des Forschungsvorhabens möglicherweise kriminelle oder andere Taten offenkundig werden, die entsprechende Maßnahmen erfordern (z.B. Untersuchung auf Drogenkonsum)?	☐	☒

2.2. Forschung an und mit vulnerablen Gruppen

	JA	NEIN
e) Sind im Forschungsvorhaben Versuchspersonen involviert, die nicht in der Lage sind, eine informierte Einwilligung[2] zu geben?	☐	☒
f) Handelt es sich um Kinder/Minderjährige?	☐	☒
g) Handelt es sich um Patient*innen?	☐	☒
h) Handelt es sich um gesunde Freiwillige für medizinische Studien?	☐	☒
i) Ist das Forschungsvorhaben mit körperlichen Eingriffen an den Studienteilnehmer*innen verbunden?	☐	☒
j) Liegen im Forschungsvorhaben Umstände vor, durch die eine Verweigerung der Teilnahme erschwert wird (z.B. Abhängigkeitsverhältnisse)?	☐	☒

3. Forschung an und mit menschlichen Zellen / Gewebe

	JA	NEIN
Planen Sie mit menschlichen Zellen oder Gewebe zu forschen?	☐	☒

4. Forschung an und mit menschlichen Embryonen / Föten

	JA	NEIN
Planen Sie an menschlichen embryonalen Stammzellen zu forschen?	☐	☒

5. Schutz personenbezogener Daten

	JA	NEIN
Werden Sie in Ihrem Forschungsvorhaben personenbezogene Daten aus eigenen oder fremden Quellen erhoben, gespeichert oder verarbeiten, die nicht anonymisiert[3] sind?	☐	☒

---

[1] Im Sinne der Grundsätze 25-32 der Deklaration von Helsinki des Weltärztebundes: https://www.bundesaerztekammer.de/fileadmin/user_upload/downloads/pdf-Ordner/International/Deklaration_von_Helsinki_2013_20190905.pdf

[2] vergl. Anmerkung 1 oder weitere Ergänzung durch https://www.forschungsdaten-bildung.de/einwilligung

[3] Für eine Einschätzung, ob es sich um ein datenschutzrelevantes Vorhaben handelt, sollte die DSGVO beachtet werden. Es findet sich dort keine Definition von **Anonymisierung**, es gibt hierzu lediglich Hinweise: „Die Grundsätze des Datenschutzes sollten […] nicht für anonyme Informationen gelten, d.h. für Informationen, die sich nicht auf eine identifizierte oder identifizierbare natürliche Person beziehen, oder personenbezogene Daten, die in einer Weise anonymisiert worden sind, dass die betroffene Person nicht oder nicht mehr identifiziert werden kann.“(Erwägungsgrund 26 DSGVO)

	JA	NEIN
**6. Menschenrechte, Nachhaltigkeit**		
Sind durch das Forschungsvorhaben und die damit gesetzten Zielen Konflikte mit Menschenrechten[4] oder Nachhaltigkeit absehbar (insbesondere im Kontext der Nachhaltigkeitsziele der Vereinten Nationen [5] oder des Übereinkommens von Paris[6])?	☐	☒
**7. Umweltschutz und Ökologie**		
a) Sind durch das Forschungsvorhaben und die damit gesetzten Zielen Konflikte mit Umwelt- und/oder Klimaschutz absehbar (insbesondere im Kontext der Nachhaltigkeitsziele der Vereinten Nationen oder des Übereinkommens von Paris)?	☐	☒
b) Beinhaltet das Forschungsvorhaben Elemente, welche die Umwelt, Tiere oder Pflanzen beeinträchtigen?	☐	☒
c) Planen Sie an oder mit geschützten Arten zu forschen?	☐	☒
**8. Technologie und Künstliche Intelligenz**		
a) Beinhaltet oder entwickelt das Forschungsvorhaben Technologien (in Hardware oder Software), von denen zu erwarten ist, dass sie ethisch relevante Entscheidungsunterstützung oder -vorbereitung[7] zur Aufgabe haben, bzw. die künftig ethisch relevante Entscheidungen treffen könnten bzw. sollen[8]?	☐	☒
b) Beinhaltet oder entwickelt das Forschungsvorhaben Technologien, von denen zu erwarten ist, dass sie die ethischen Prinzipien der Achtung der menschlichen Autonomie, Schadensverhütung, Fairness und Erklärbarkeit[9] verletzen können?	☐	☒

Pseudonymisierung ist die Verarbeitung personenbezogener Daten in einer Weise, dass die personenbezogenen Daten ohne Hinzuziehung zusätzlicher Informationen nicht mehr einer spezifischen betroffenen Person zugeordnet werden können, sofern diese zusätzlichen Informationen gesondert aufbewahrt werden und technischen und organisatorischen Maßnahmen unterliegen, die gewährleisten, dass die personenbezogenen Daten nicht einer identifizierten oder identifizierbaren natürlichen Person zugewiesen werden (Art 4 DSGVO).

[4] auswaertiges-amt.de/blob/209898/beeab63c2704f684c606a65589cf236c/allgerklaerungmenschenrechte-data.pdf

[5] https://sdgs.un.org/goals

[6] Europäische Union: Übereinkommen von Paris. Amtsblatt der Europäischen Union L 282/4, Brüssel (2016). https://eur-lex.europa.eu/legal-content/DE/TXT/PDF/?uri=CELEX:22016A1019(01)

[7] z.B. auf der Basis großer Datenmengen, Sensordaten, Bildern.

[8] z.B. durch Algorithmen, die auf Künstlicher Intelligenz (KI) z.B. Machine Learning (ML) beruhen.

[9] https://www.demographie-netzwerk.de/site/assets/files/5064/ethicsguidelinesfortrustworthyai-depdf.pdf

Frage	JA	NEIN
**9. Länder mit niedrigem und mittlerem Einkommen**		
a) Werden für das Forschungsvorhaben Ressourcen aus Ländern mit niedrigem oder mittlerem Einkommen genutzt, ohne dass ein adäquater Benefit-Ausgleich eingeplant ist?	☐	☒
b) Werden bei dem Forschungsvorhaben Daten in Ländern mit niedrigem oder mittlerem Einkommen erhoben, ohne dass Forschenden aus den betroffenen Ländern Zugang zu den Daten und Forschungsergebnissen gewährt wird?	☐	☒
**10. Dual-Use**		
a) Gibt es Bedenken oder eine nicht nur geringe Wahrscheinlichkeit, dass die Forschungsergebnisse für militärische Zwecke genutzt werden können?	☐	☒
b) Beinhaltet das Forschungsvorhaben Dual-Use-Güter (Güter mit doppeltem Verwendungszweck gemäß EG-Verordnung 428/2009, d.h. Güter, die einschließlich Datenverarbeitungsprogramme und Technologie sowohl für zivile als auch für militärische Zwecke verwendet werden können)?	☐	☒
c) Beinhaltet das Forschungsvorhaben Kooperationspartner aus dem nicht-zivilen Bereich und kann ein ausschließlich ziviler Charakter des Vorhabens nicht eindeutig belegt werden?	☐	☒
**11. Missbrauch**		
Hat Ihre Forschung ein Potenzial für den Missbrauch von Forschungsergebnissen[10]?	☐	☒

Hiermit bestätige ich, dass ich die Fragen wahrheitsgemäß und nach bestem Wissen und Gewissen beantwortet habe.

16.12.2021 Pascal Vögele

*Datum Unterschrift Antragsteller*in*

*(b[illegible]beiten auch Unterschrift Betreuer*in)*

[10] z.B.: Könnten die Materialien / Methoden / Technologien und das Wissen, das oder die erzeugt wurden, Menschen, Tieren, Pflanzen oder der Umwelt schaden, wenn sie verändert oder weiterentwickelt werden?
